# Clinical Pharmacists on Medical Care of Pediatric Inpatients: A Single-Center Randomized Controlled Trial

**DOI:** 10.1371/journal.pone.0030856

**Published:** 2012-01-23

**Authors:** Chuan Zhang, Lingli Zhang, Liang Huang, Rong Luo, Jin Wen

**Affiliations:** 1 The Department of Pharmacy, West China Second University Hospital, Sichuan University, Chengdu, China; 2 The Department of Pediatrics, West China Second University Hospital, Sichuan University, Chengdu, China; 3 The Chinese Cochrane Centre/Chinese Evidence-based Medicine Centre, West China Hospital, Sichuan University, Chengdu, China; Genentech Inc., United States of America

## Abstract

**Objective:**

To explore the best interventions and working patterns of clinical pharmacists in pediatrics and to determine the effectiveness of clinical pharmacists in pediatrics.

**Methods:**

We conducted a randomized controlled trial of 160 pediatric patients with nerve system disease, respiratory system disease or digestive system disease, who were randomly allocated into two groups, with 80 in each group. Interventions by clinical pharmacists in the experimental group included answering questions of physicians and nurses, giving advice on treating patients, checking prescriptions and patient counseling at discharge. In the control group, patients were treated without clinical pharmacist interventions.

**Results:**

Of the 109 interventions provided by clinical pharmacists during 4 months, 47 were consultations for physicians and nurses, 31 were suggestions of treatment, with 30 accepted by physicians (96.77%) and 31 were medical errors found in 641 prescriptions. Five adverse drug reactions were submitted to the adverse drug reaction monitoring network, with three in the experimental group and two in the control group. The average length of stay (LOS) for patients with respiratory system diseases in the experimental group was 6.45 days, in comparison with 10.83 days in the control group, which was statistically different (*p* value<0.05); Average drug compliance rate in the experimental group was 81.41%, in comparison with 70.17% of the control group, which was statistically different (*p* value<0.05). Cost of drugs and hospitalization and rate of readmission in two weeks after discharge in the two groups were not statistically different.

**Conclusion:**

Participation by clinical pharmacists in the pharmacotherapy of pediatric patients can reduce LOS of patients with respiratory system disease and improve compliance rate through discharge education, showing no significant effects on prevention of ADR, reduction of cost of drugs and hospitalization and readmission rate in two weeks.

**Trial Registration:**

Chinese Clinical Trial Registry ChiCTR-TRC-10001081

## Introduction

Many drugs used in children are done so unlicensed, off-label, unsafely or without any evidence of efficacy in children [1]. A report of WHO in 2005 showed three times more medical errors in children than in adults [2]. Suitable pediatric formulations and doses in children are required, especially for drugs with a narrow therapeutic index, which leads to serious morbidity or mortality when used at a 10-fold dosage [3]. Thus greater focus on improvement of safety of drugs used in children is necessary.

It was reported that clinical pharmacist participation in the medical care of patients could improve the safety of drugs [4]–[5]. Having a pharmacist on a rounding team in an intensive care unit (ICU) has been shown to reduce the incidence of adverse drug events (ADEs) by two thirds [6]. A prospective study by Fortescue et al. showed that ward-based clinical pharmacists prevented 81% of potentially harmful medication errors [7]. Thus, clinical pharmacists can not only improve drug safety, but also serve to lower costs [8], improve quality of pharmacotherapy [9], coordinate the relationship of Pharmacy with other departments [10] and enrich patient drug knowledge [11].

Nevertheless, it is difficult to determine the important role of clinical pharmacists in pediatrics. Studies by Devlin [12] and Dice [13] on the effects of participation of clinical pharmacists in treatment of Total Parenteral Nutrition (TPN) showed that pharmacist-monitored TPN proved cost effective in comparison with the standardized solution without pharmacist monitoring. However, a study by Gibson demonstrated that the results of clinical pharmacists' involvement in pharmacotherapy [14] were not statistically different. A systematic review of 18 observational studies by Navneet [15] highlighted the importance of pharmacists to medicine management in pediatric patients, but there have not been any randomized controlled trials so far [15].

We conducted a randomized controlled trial in wards of pediatric patients with common diseases to answer the following questions:

1. What types of interventions do clinical pharmacists provide?
2. Can participation of clinical pharmacists in the medical care of inpatients reduce the cost of drugs and hospitalization, LOS and readmission rate and improve patient compliance rate after discharge?

## Methods

The protocol for this trial and supporting CONSORT checklist are available as supporting information; see Checklist S1 and Protocol S1. The trial was approved by Ethics Committee of West China Second University Hospital, Sichuan University and registered in Chinese Clinical Trial Registry and the registered number was ChiCTR-TRC-10001081. Patients had given their written informed consents.

### 2.1 Study site location and participants

This randomized controlled trial was conducted at West China Second University Hospital, Chengdu, Sichuan, China, from December 1, 2010, through to March 31, 2011. Eligible participants were pediatric patients with nerve system disease, respiratory system disease, or digestive system disease, whose age ranged from birth to 18 years old. “Nerve system disease” defined as epilepsy, cephalomeningitis, intracranial infection, hyperspasmia, infantile spasms; “Respiratory system disease” defined as infection of upper respiratory tract, pneumonia and bronchitis; “Digestive system disease” defined as diarrhiea, gastrointestinal hemorrhage, esophagitis, gastritis, enteritis. Every patient or patient's family was asked to give written informed consent. Those who were critical or could not speak Chinese or whose families declined to participate in the trial were excluded.

### 2.2 Study design

#### 2.2.1 Randomization and concealing allocation

Randomization was completed by SPSS 16.0-generated algorithm. Treating assignments, kept in sealed opaque envelopes with only number labeled, were opened after patient gave their informed consents. One of the two clinical pharmacists distributed envelopes and recorded patients in each group enrollment and patient assignment.

#### 2.2.2 Blinding

Patients and clinical pharmacists were aware of the interventions while trial research assistants and statisticians, responsible for outcome recording and data analysis, respectively, were blinded to treatment assignment.

#### 2.2.3 Follow up

The protocol planned that patients were interviewed 3 or 4 days after discharge. But the compliance rate is connected with whether the drug courses have finished. Therefore, time of follow-up was determined by how long the discharge drugs were used. Patients were usually interviewed on phone when discharge drugs were half finished.

### 2.3 Clinical pharmacists' interventions

#### 2.3.1 Experimental group

In the morning shift, clinical pharmacists made rounds together with doctors in charge and provided interventions, which included an assessment of the patients' medication, diagnosis, experimental index and drug treatment. Additionally, they gave advice on drug selections in view of therapeutic guidelines, the national essential medicine list and national basic insurance medicine catalogs, provided pharmacokinetic consultations and drug information for physicians and nurses, checked prescriptions and communicated with physicians about any medication errors, reviewed the indications, directions for use, and possible adverse effects of each discharge medication and gave discharge education to patients.

#### 2.3.2 Control group

Patients randomized to the control group were treated following the traditional medical model, in which physicians and nurses were responsible for treatment and clinical pharmacists were excluded from pharmacotherapy and discharge education.

### 2.4 Primary outcomes

#### 2.4.1 Interventions by clinical pharmacists

Three types of interventions were documented by two clinical pharmacists: 1. answering questions of physicians and nurses; 2. suggestions of treatment; 3. prevention of medication errors. Answering the questions of physicians and nurses meant that clinical pharmacists gave an accepted answer to questions related to drugs by physicians and nurses. Suggestion of treatment referred to advice by clinical pharmacists on patient's treatment which was adopted by physicians. Prevention of medication errors meant that clinical pharmacist checked the prescriptions and noted the medication errors which were corrected by physicians. Of the 109 interventions, 46 were on antibiotics, accounting for 42.2%; 21 on drugs for digestive system, 18 on drugs for respiratory system, 13 on drugs for nerve system, 4 on glucocorticoid, 4 on drugs for dermatopathya, 2 on electrolyte and 1 on drug for hematologic system. This measurement was not only the different interventions between experimental group and controlled group but also the crucial factor relative to repeatability and reproducibility of this trial. The clinical pharmacists did not participate in the treatment of patients in controlled group, so we only recored the interventions in patients in experimental group.

#### 2.4.2 The number of adverse drug reactions (ADR)

Clinical pharmacists detected ADRs in the experimental group while the physicians and nurses detected that in the control group.

#### 2.4.3 Length of stay

Length of stay (LOS) refers to the number of days staying in hospital from admission to discharge.

#### 2.4.4 Cost of drugs

Cost of drugs includes total charges of Western medicines and Chinese traditional medicines.

#### 2.4.5 Cost of hospitalization

Cost of hospitalization is defined as total charges in the hospital, including cost of drugs, examining and nursing care.

### 2.5 Secondary outcomes

#### 2.5.1 Compliance rate

The method by Williford and Johnson [16] was adopted in terms of measurement of compliance rate. In accordance with the time set in the method, clinical pharmacists interviewed patients on phone in the follow-up, asking the following questions: 1. to state the name of each medicine prescribed at discharge; 2. to state the therapeutic action of each medicine; 3. to state the usage of each medicine at discharge; and 4. to state the administration of the drug taken at present
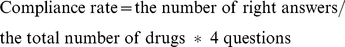



#### 2.5.2 Readmission rate

Readmission rate referred to the proportion of patients readmitted to hospital in two weeks after discharge of the total patients.

### 2.6 Statistical analysis

Intention to treat (ITT) was used to analyze the data. Statistical software SPSS 16.0 was used for all analyses. Continuous parametric length of stay, compliance rate and readmission rate were analyzed by student's t-test. The values, like age of patients, cost of drugs and hospitalization, which were not normal distribution were analyzed by nonparametric test Wilcoxon rank sum test. Discrete data was analyzed with chi-square test. Results with a p value<0.05 were considered statistically significant. Subgroup analysis was adopted for LOS according to different types of diseases such as nerve system disease, digestive system disease and respiratory system disease. Multiple linear regression analysis was used to explore the relationship between compliance rate and age of patients, number of discharge drugs and time of follow up. Of the seven indexes, interventions of clinical pharmacists were descriptive index, and thus could not be counted in as samples. While it was reported in some literatures that rate of ADR decreased in the group with clinical pharmacists, other studies found that with interventions of clinical pharmacists, the rate increased. Thus, We could not determine whether interventions of clinical pharmacists could decrease the rate of ADR It was the same case for the length of stay; in terms of cost of drugs and hospitalization,which was nonparametric distribution data, they were lack of mean value. Therefore, compliance rate alone was used to power calculation.

Compliance rate was reported to be 75% in the literatures [16]–[17]. In our study, clinical pharmacists in pediatric unit were trying to improve the compliance rate to 95%. In order to achieve such a result, we set a power of 90% and the 5% level of significance (α = 0.05, β = 0.1). Through calculation, we found that this required 67 patients in both groups. With 20% loss of follow-up considered, a total of 160 patients were required, with 80 in each group.

## Results

**Figure 1:**
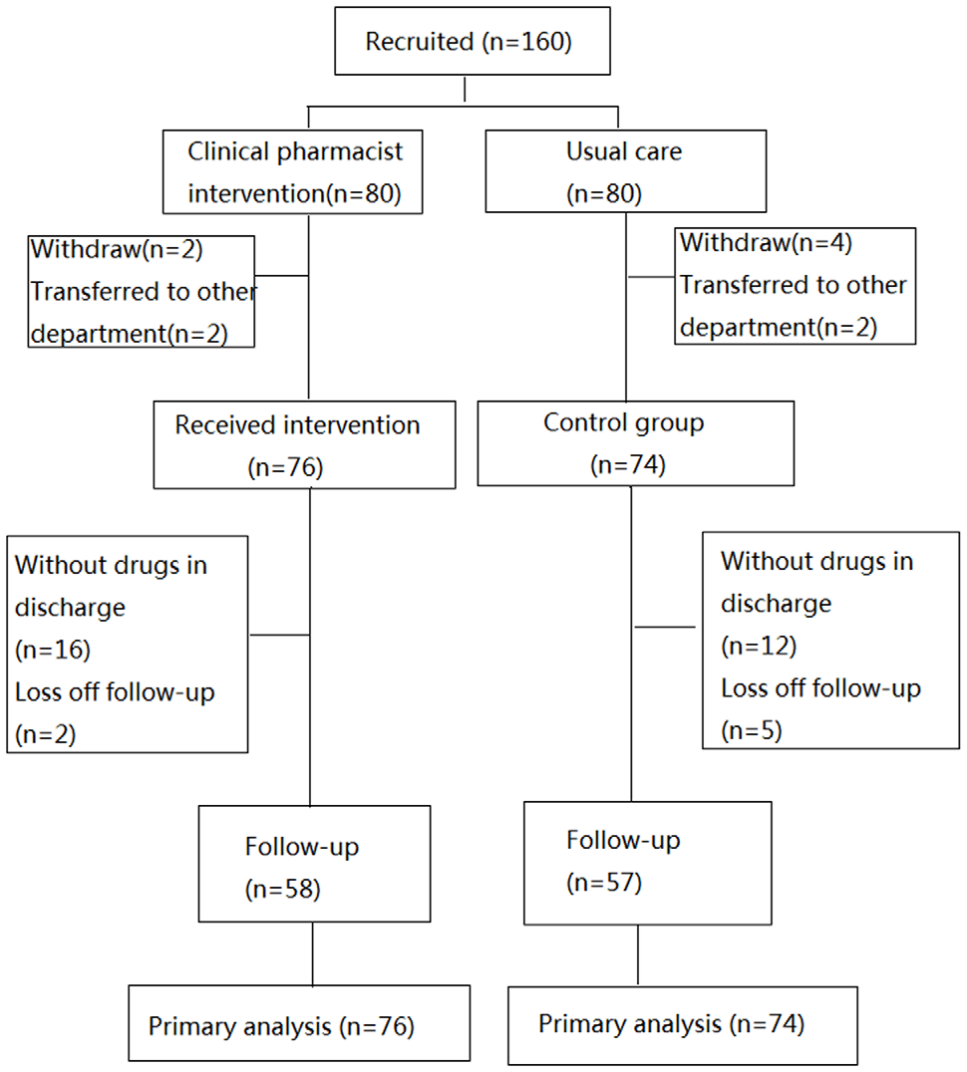
Trial flow chart.

We recruited 160 patients to this study. Inclusion procedures are illustrated in Figure 1. The 160 patients were randomly allocated to the experimental group and the control group, with 80 in each group. In the experimental group, after two patients gave up treatment and another two were transferred to other department, only 76 patients received clinical pharmacists' intervention. In the control group, after four patients gave up treatment and another two were transferred to other department, only 74 patients received usual care. In the phase of follow-up, 16 experimental patients were discharged without drugs and 2 could not be contacted, so only 58 received an interview. Comparatively, 12 patients from the control group were discharged without drugs and 5 could not be contacted, so 57 received an interview. Trial flow chart is presented in figure 1.

Characteristics of patients are presented in Table 1. There were no significant differences between the two groups.

**Table 1:** Characteristics of patients.

Characteristic	Experimental group( = 80)	Control group (n = 80)	*P* value
Age, No.y			
<1	20	21	0.941
1∼5	36	34	
5∼10	13	12	
>10	11	13	
Sex, No.(M/F)	43/37	44/36	0.874
Disease			0.637
Nerve system disease	36	42	
Respiratory system disease	29	25	
Digestive system disease	15	13	
weight(kg)	15.86±10.73	16.16±11.33	0.864
Temperature(°C)	36.93±0.93	36.92±0.72	0.909
Pulse(sequence/min)	115±22.84	116±23.25	0.646
Breath(sequence/min)	28.2±7.76	29.2±8.87	0.444

### 3.1 Primary outcomes

#### 3.1.1 Interventions by clinical pharmacists

Clinical pharmacists provided 107 interventions. These included 47 questions asked by physicians or nurses, 31 suggestions of treatment and the prevention of 31 medication errors (Table 2). During the trial, the 47 questions for clinical pharmacists posed by doctors and nurses could all be classified into 7 categories: information of drug production, dosage and administration, specification, pharmacological actions, management, interaction and contraindication. Of all the questions, the greatest number were concerned about dosage and administration, accounting for 55.3%, followed by those about pharmacological actions, for 25.5% and 8.5% questions were about contraindications. Of the 31 suggestions, 64.52% were on drugs selection or discontinuance and 16.13% on drugs combinations. 71% of the 31 suggestions were on use of antibiotics. Of the 683 prescriptions checked by clinical pharmacists, 31 medication errors were found with an incidence frequency of 4.53%.

**Table 2:** Interventions by clinical pharmacists.

Interventions	No. (%)
**1.Answering the question of physicians or nurses (47)**	
Information of drug production	1 (2.1%)
Drugs dosage and usage	26 (55.3%)
Drugs specification	1 (2.1%)
Drugs pharmacology	12 (25.5%)
Drugs management	1 (2.1%)
Drugs interaction	2 (4.2%)
Drugs contraindication	4 (8.5%)
**2.Suggestion of treatment (31)**	
Drugs combination	5 (16.13%)
Dosage to add or subtract	3 (9.67%)
Selection or discontinuance drugs	20 (64.52%)
Time of usage	1 (3.22%)
Drug formulation	1 (3.22%)
Prevention of ADR	1 (3.22%)
**3.Prevention of medication errors (31)**	
Prescription errors	12 (38.71%)
Dosage errors	4 (12.91%)
Preparation errors	10 (32.26%)
Technology errors	1 (3.2%)
Compliance errors	4 (2.90%)

#### 3.1.2 The number of adverse drug reaction (ADR)

Five ADRs were identified in the study, with three in the experimental group and two in the control group. Of the three in the experimental group, one patient developed rash after taking rifampicin, another patient's neutrophile granulocyte count began to drop after taking Ganciclovir, and the third suffered emesis after taking oral cough syrup. Of the two patients in the control group, one was reported to have developed rashes after injection of tazobactam sodium and the other one was agitated after taking oral ammonia bromine. The five patients recovered after discontinuing these drugs. They were submitted to the adverse reaction monitoring network by clinical pharmacists.

#### 3.1.3 Length of stay

76 patients in the experimental group and 74 patients in the control group were analyzed with respect to LOS. The result showed that mean of LOS in the two groups was 7.33 and 9.06 days respectively, which was statistically different (Table 3).

**Table 3:** LOS in the two groups.

	Experimental group		Control group		P value
	No.	LOS,D	No.	LOS,D	
Total patients	76	7.33±3.52	74	9.06±5.47	0.02
*Subgroup analysis*					
Nerve system disease	33	8.36±4.15	38	9.76±6.32	0.28
Respiratory system disease	29	6.45±2.91	24	10.83±6.72	0.003
Digestive system disease	14	6.71±2.43	12	6.08±3.11	0.57

Sub-analysis showed that in the patients with nerve system disease and digestive system disease, LOS in the two groups was not statistically different (Table 3) while concerning patients with respiratory system diseases, LOS was statistically different (Table 3).

#### 3.1.4 Cost of drugs and hospitalization

Through comparison of cost of drugs and hospitalization between 76 patients in the experimental group and 74 patients in the control group, a rank sum test demonstrated that cost of drugs and hospitalization in the two groups were not statistically different (Table 4).

**Table 4:** Cost of drugs and hospitalization in the two groups.

	Intervention group (n = 76)	Control group (n = 74)	P value
Cost of drugs (RMB, yuan)			
<1000	36	35	0.945
1000∼3000	24	26	
3000∼5000	10	4	
>5000	6	9	
Cost of hospitalization (RMB, yuan)			
<2000	18	15	0.125
2000∼4000	33	25	
4000∼6000	10	10	
>6000	15	24	

### 3.2 Secondary outcomes

In the follow-up, a total of 58 and 57 patients were included into the experimental and the control group, respectively. A significant improvement of compliance rate was seen in the experimental group, in which clinical pharmacists gave discharge education to patients. Multiple linear regression analysis showed that drug compliance rate was not related to age, number of drugs, or time of follow-up, as these showed no statistical differences (Table 5).

**Table 5:** Secondary study outcomes.

	Intervention Group (n = 58)	Control group (n = 57)	P value
**Baseline**			
Discharge drugs, No.	2.74±1.58	2.64±1.41	0.742
Time of follow-up, day	3.69±1.25	3.96±1.27	0.243
**Compliance rate (%)**	81.41±19.42	70.17±22.33	0.005
**Readmission rate, No.(%)**	4 (9.1%)	5 (13.2%)	0.726

We first compared the number of discharge drugs and time of follow-up in the two groups with consideration to their relevance to drug compliance rate. The result showed that the average number of discharge drugs in the two groups was 2.74 and 2.64 respectively, which was not statistically different (*P* value>0.05) and that the time interval from discharge to follow-up in the two groups was 3.69 days and 3.96 days, with a *P* value>0.05 showing no statistical differences. There were no significant differences between the two groups (Table 6). Mean of compliance rate was 81.41% in the experimental group, and 70.17% in the control group, which was statistically different (*P* value<0.05, Table 6).

**Table 6:** Coefficients.

Variable	Unstandardized Coefficients		Standardized Coefficients	t	P
	B	Std. Error			
Constant	86.339	7.072		12.208	0.000
Age	−0.041	0.042	−0.092	−0.984	0.327
No. of drugs	−2.082	1.373	−0.144	−1.517	0.132
Time of follow-up	−0.795	1.633	−0.046	−0.487	0.627

Comparing the readmission rate of patients with respiratory diseases or digestive system diseases between the experimental group (44 cases) and the control group (38 cases), showed that there were 4 patients in the experimental group (9.1%) and 5 patients in the control group (13.2%) that needed outpatient treatment within 2 weeks after discharge, which was not statistically different (P value>0.05, Table 5). This result suggested that interventions of clinical pharmacists in pharmacotherapy could not reduce rate of readmission.

## Discussion

This study is the first randomized controlled trial on the effectiveness of clinical pharmacists in pediatric hospitalized patients. It showed that on the one hand interventions by clinical pharmacists on pharmacotherapy of pediatric patients were associated with a significantly shorter LOS and a higher compliance rate after hospital discharge. On the other hand, no differences were seen in rate of ADR, cost of drugs and hospitalization or readmission rate. The shorter LOS in patients of the experimental group, especially in the patients with respiratory system disease, may be attributed to suggestions of treatment, and higher compliance rate after discharge was a result of discharge education provided by clinical pharmacists.

Clinical pharmacists included into our study had fixed work hours. In the morning shift, they became familiar with history of present illness and drugs taken for newly admitted patients. Then they made rounds with the pediatric team, provided suggestions of treatment, checked the prescriptions of physicians, gave consultations to discharged patients. Afterwards, they stood by for calls throughout the day. The schedule of clinical pharmacists in the study was similar to that reported by Leape [18] which investigated pharmacist participation on physician rounds in the intensive care unit. In their study, Krupicka et al. reported that clinical pharmacists worked for 5 days a week with weekend services provided in a centralized location. The clinical pharmacists attended morning rounds at least twice per week [19], which was less than that in our study.

Percentage distribution of clinical pharmacists' time in each intervention was not calculated in our study. However, it was estimated that clinical pharmacists spent 80% of their working time in providing suggestions, checking the prescriptions, doing rounds of the ward and giving consultations to the patients. Gibson [9] reported on distribution of time use by clinical pharmacists in a pediatric unit: checking doses in patient trays for correctness and new physician's orders covered 17.5%, respectively; 12.5% time was spent in reconciling pharmacy patient profile with nursing, reference research for drug information, checking unit dose injections and nursing station visits and supplying first doses covered 10%, answering questions from nursing staff, reading patient medical records, calculating pediatric doses, and dispersing prescriptions for patient discharge covered 5%. Only 2.5% time was spent in providing suggestions for doctors.

109 interventions were documented in 4 months in our study, comparatively less than other studies completed abroad. Kucukarslan et al. [20] reported that more than 150 interventions were provided by clinical pharmacists in 3 months. Koren et al. [21] documented 390 interventions conducted within 2 months in her studies while Strong and Tsang [22] provided 361 interventions within 2 weeks.

Two factors could be proposed to explain these findings. First, other studies included more patients than our study did. For example, pharmacist: patient ratio was approximately 1∶15 in Kucukarslan et al.'s study [20]. Second, clinical pharmacists in our study spent less time on clinical treatment, Kucukarslan et al. and Krupicka et al. documented all interventions that occurred during the shift from 7:00 am to 3:30 pm [19], [20], while clinical pharmacists in our study were available on call in the afternoon.

Among the 31 suggestions made by clinical pharmacists, 30 (96.77%) were accepted by the treating physicians. Similarly, of the 48 interventions recorded by Virani and Crown [23], 47 were adopted. Condren et al. [24] reported that during 12 months 91% of 4605 interventions performed for 3978 patients were accepted by the physicians. A lower acceptance rate of 80% was reported in a study by Guy et al. [25]. These findings demonstrated that reasonable suggestions provided by clinical pharmacists are generally accepted by physicians.

During the trial, 31 medical errors in 684 prescriptions were detected by clinical pharmacists. The frequency of errors was 4.53%, which was higher than other studies. A study conducted by Folli et al. [26] in two large children's hospitals in the US found the frequency of errors was 4.9 and 4.5 errors per 1000 medication orders. Similar to our study, the frequency of order errors declined as physician training status increased. The frequency of errors in Riley Hospital for Children and Indiana University Hospital was 2.66% (1277/48034) and 1.34% (1012/75333), respectively. The most common types of errors in our study were prescription and preparation errors while the most common types in other studies were wrong dose and inappropriate dosage schedule [19], [22].

Of the five ADR, four were detected in the hospital, and one was detected during follow up. The incidence rate of ADR, 3.33%, was less than the overall, 6.20%, as reported in a systematic review [27]. This suggests that more attentions should be paid to ADRs in children.

Like the other three studies, our findings demonstrate the significant role of clinical pharmacists in reduction of LOS of patients. The three randomized trials demonstrated that participation by clinical pharmacists in pharmacotherapy could reduce length of stay by 1.3 days (18.05%), to 4.4 days (30.35%), which was statistically significant [28]–[30]. One controlled study on a large sample (n = 7219) showed that clinical pharmacists could reduce LOS by 2.4 days (18.18%), which was statistically different [31]. A retrospective study showed that clinical pharmacists reduced LOS by 5.99 days (31.39%), but in the study only 23 cases were included as interventions [32]. Nevertheless, some studies still show that participation of clinical pharmacists in pharmacotherapy does not cause a statistically significant decrease in LOS [33]–[37]. Research by Lal, Anassi and McCants [10] demonstrated that clinical pharmacists participating in pediatric pharmacotherapy conducted 504 interventions, which reduced LOS from 4.38 days to 4.26 days. Through analysis of results in the subgroup, we found that with participation of clinical pharmacists in treatment of patients with respiratory diseases LOS in the two groups was statistically different, while in treatment of patients with neurological and digestive diseases, LOS was not statistically different. The result may be caused by the fact that over 50% of the more than 100 interventions were related to drugs on respiratory system and that most treatment suggestions were on the use of antibiotics.

With different costs of treatment for different diseases, costs of drugs and hospitalization in the study differed from a normal distribution. Results by nonparametric test showed no significant differences. Conversely, research abroad demonstrated that participation of clinical pharmacists in treatment could reduce costs of drugs and hospitalization and that there were significant differences [38]. A study by Lal, Inassi and McCant [10] on pediatric clinical pharmacists showed that with clinical pharmacists' participation, the cost of treatment of a pediatric inpatient during 6 months had been reduced by $7227. 83, which significantly demonstrated the economical efficiency of participation of clinical pharmacists in drug treatment of patients.

Failure to comply with medication instructions commonly led to serious adverse outcomes, and noncompliance after discharge may place patients at risk for suboptimal response and toxic outcomes, including readmission to the hospital [39]–[41]. Patient education before discharge has been recognized a valuable method to improve compliance of patients [42], [43]. Our study demonstrated that patient education could increase the compliance rate by 16.02%, which was close to the results of the other three randomized controlled trials [44], [45].

Unexpectedly, we did not find any linear relationship between compliance rate and the age of patients, time of follow-up and number of discharge drugs. Other studies demonstrated a major factor associated with noncompliance in discharged patients was the complexity of the prescribed regimen [16], [39]. Several reasons could be considered. First, the people interviewed in follow-up were families of patients. Secondly, the time of follow-up was fixed, approximately 3 to 4 days after discharge. Thirdly, most patients were prescribed comparatively fewer drugs upon discharge, from one to six.

Readmission rate in two weeks after discharge in our study was 9.1% and 13.2% in experimental and control group, respectively. Different readmission rates have been reported in different diseases, from 4.58% to 39% [30], [46]–[47]. Different time of follow-up was also associated with readmission rate. Al Rashed [48] reported that readmission rate in two or three weeks after discharge was 11.6% in experimental group and 32.5% in the control group. There was significant difference between the two groups. No significant difference was showed in the readmission rate at 30 days or 3 months after discharge in both groups [46], [47].

Seasonal bias was little in our trial. Our trial lasted five months, (December 2010—April 2011), from winter to spring. We investigated the diseases of patients in the department for incidence of diseases in summer and autumn. The result showed that there were 374 cases in summer and 363 ones in autumn. Of the cases in summer, 194 were nerve system disease, 112 respiratory system diseases, and 66 digestive system disease. Comparatively, of the cases in autumn, 198 were nerve system diseases, 86 respiratory system diseases and 79 digestive system diseases. Incidences of diseases of nerve system, respiratory system and digestive system in the four seasons were not statistically different. *P* = 0.114.

There are still limitations in our study. First, clinical pharmacists did not participate in treatment of patients in the control group, but in fact, clinical pharmacists also provided suggestions for patients in the control group when the physicians consulted them, which led to contamination and interference of results of the two groups. Second, the clinical pharmacists also participated in treatment of patients with serious disease who were excluded in the trial, but the interventions were not documented. Therefore, the number of interventions was more than what were recorded in this trial. Third, the power of test in patients with nerve system disease and digestive system disease were 0.29 and 0.1 respectively, indicating that the sample size was not enough. Nevertheless, as this trial was only an exploratory study, we will expand the sample size in the subsequent studies to observe whether difference exists

### Conclusion

1. The participation of clinical pharmacists in pharmacotherapy of pediatric patients can reduce length of stay of patients with respiratory system disease and improve compliance rate through discharge education.
2. Our study did not find significant effect of clinical pharmacists on prevention of ADR, reduction of cost of drugs and hospitalization or readmission rate in two weeks.

## Supporting Information

Protocol S1 **Clinical Pharmacists on Medical Care of Pediatric Inpatients: a Randomized Controlled Trial Protocol.**
(DOC)

Checklist S1 **CONSORT 2010 checklist contained in the article.**
(DOC)
