# Supplementary material for: Clinical Pharmacists on Medical Care of Pediatric Inpatients: A Single-Center Randomized Controlled Trial
Source: PLoS One. 2012 Jan 23;7(1):e30856. doi: 10.1371/journal.pone.0030856 (PMC3264625; doi:10.1371/journal.pone.0030856)
Supplement: Protocol S1 — Clinical Pharmacists on Medical Care of Pediatric Inpatients: a Randomized Controlled Trial Protocol. (DOC) [file pone.0030856.s001.doc]

**Protocol S1**

**Clinical Pharmacists on Medical Care of Pediatric Inpatients: a Randomized Controlled Trial Protocol**

A systematic review of 18 observational studies by Navneet highlighted the importance of pharmacists to medicines management in pediatric patients. But there had not been any randomized controlled trial so far.

We will conduct a randomized controlled trial in wards of pediatric patients with predominant diseases to resolve the following problems:

1. What types of interventions do clinical pharmacists provide?
2. Can participation of clinical pharmacists in medicare of inpatients reduce cost of drugs and hospitalization, LOS and readmission rate and improve compliance rate of patients after discharge?

**Study site location and participants** This randomized controlled trial will be conducted at West China Second University Hospital, Chengdu, Sichuan, China. Eligible participants are pediatric patients with nerve system disease, respiratory system disease, or digestive system disease, whose age ranged from birth to 18 years old. Patients with the following conditions are excluded: 1. patients’ family rejects to participate in the trial; 2. patients with serious disease; 3. patients’ family can not communicate with the researchers because of language barriers. Every patient or patient’s family in this trial will be required to give written informed consent.

**Power calculation** Compliance rate was reported to be 75% in the literatures. In our study, clinical pharmacists in pediatric unit are trying to improve the compliance rate to 95%. In order to achieve such a result, we set a power of 90% and the 5% level of significance (α=0.05，β=0.1). Through calculation, we find that it requires 67 patients in both groups. With 20% loss of follow-up considered, a total of 160 patients are required, with 80 ones in each group.

**Randomization and concealing allocation** Randomization will be completed by SPSS 16.0-generated algorithm. Treating assignments, kept in sealed opaque envelopes with only number labeled, are opened after patients give their informed consents. One of the two clinical pharmacists distributes envelopes and records patients in each group enrollment and patient assignment.

**Blinding** Research assistant of trial and statisticians who are responsible for outcome recording and data analysis respectively are blinded to treatment assignment.

**Follow up** Patients will be interviewed on phone when 3 or 4 days after discharge .

**Intervention:**

*Experimental group* In the morning shift, clinical pharmacists will make rounds together with doctors in charge and provide interventions. They provide pharmacokinetic consultations and drug information for physicians and nurses, check prescriptions and communicate with doctors if any medication errors, review the indications, directions for use, and possible adverse effects of each discharge medication and give discharge education to patients.

*Control group* Patients in the control group will be treated following the traditional medical model, in which clinical pharmacists are excluded from pharmacotherapy and discharge education.

**Measurement**

**Primary outcomes**

**Interventions by clinical pharmacists** Three types of interventions will be documented by two clinical pharmacists: 1. answering questions of physicians and nurses; 2. suggestions of treatment; 3. prevention of medication errors.

**The number of adverse drug reaction (ADR)** Clinical pharmacists detect the number of ADR in the experimental group while the physicians and nurses detect that in the control group.

**Length of stay** Length of stay (LOS) refers to the number of days staying in hospital from admission to discharge.

**Cost of drugs** Cost of drugs includes total charges of west medicines and Chinese traditional medicines.

**Cost of hospitalization** Cost of hospitalization is defined as total charges in the hospital, including cost of drugs, examining and nursing care.

**Secondary outcomes**

**Compliance rate** Clinical pharmacists will interview patients on phone in the follow-up, asking the following questions: 1. to state the name of each medicine prescribed at discharge; 2. to state the therapeutic action of each medicine; 3. to state the usage of each medicine at discharge; and 4. to state the administration of the drug taken at present

Compliance rate= the number of right answers / the total number of drugs * 4 questions

**Readmission rate** Readmission rate refers to the proportion of patients readmitted to hospital in two weeks after discharge of the total patients.

**Statistical analysis** Statistical software SPSS 16.0 was used for all analyses. Quantitative data were presented in mean value ± standard deviation data and enumeration data were presented in ratio. With a p value<0.05, the result was statistically different. Multiple linear regression analysis was used to explore the relationship between compliance rate and age of patients, number of discharge drug and time of follow up.
